# Marine Derived Polysaccharides for Biomedical Applications: Chemical Modification Approaches

**DOI:** 10.3390/molecules13092069

**Published:** 2008-09-03

**Authors:** Giovanna Gomez d’Ayala, Mario Malinconico, Paola Laurienzo

**Affiliations:** Istituto di Chimica e Tecnologia dei Polimeri, C.N.R.-Via Campi Flegrei, 34- 80078 Pozzuoli (Naples), Italy

**Keywords:** Alginate, chitin, chitosan, chemical modification

## Abstract

Polysaccharide-based biomaterials are an emerging class in several biomedical fields such as tissue regeneration, particularly for cartilage, drug delivery devices and gel-entrapment systems for the immobilization of cells. Important properties of the polysaccharides include controllable biological activity, biodegradability, and their ability to form hydrogels. Most of the polysaccharides used derive from natural sources; particularly, alginate and chitin, two polysaccharides which have an extensive history of use in medicine, pharmacy and basic sciences, and can be easily extracted from marine plants (algae kelp) and crab shells, respectively. The recent rediscovery of poly-saccharide-based materials is also attributable to new synthetic routes for their chemical modification, with the aim of promoting new biological activities and/or to modify the final properties of the biomaterials for specific purposes. These synthetic strategies also involve the combination of polysaccharides with other polymers. A review of the more recent research in the field of chemical modification of alginate, chitin and its derivative chitosan is presented. Moreover, we report as case studies the results of our recent work concerning various different approaches and applications of polysaccharide-based biomaterials, such as the realization of novel composites based on calcium sulphate blended with alginate and with a chemically modified chitosan, the synthesis of novel alginate-poly(ethylene glycol) copolymers and the development of a family of materials based on alginate and acrylic polymers of potential interest as drug delivery systems.

## Contents

1. Introduction
2. Polysaccharides as biomaterials
3. Alginate: structure and chemical modification
4. Alginate-based materials for drug-delivery applications
5. Alginate for cell immobilization
6. Chitin and chitosan: structure and chemical modification
7. Composites and hydrogels based on *N*-succinylchitosan/alginate blends
8. Conclusions

## 1. Introduction

By far the majority of carbohydrate materials in Nature occur in the form of polysaccharides. By our definition, polysaccharides include not only those substances composed only of glycosidically linked sugar residues, but also molecules that contain polymeric saccharide structures linked via covalent bonds to amino acids, peptides, proteins, lipids and other structures. 

Polysaccharides, also called glycans, consist of monosaccharides and their derivatives. If a polysaccharide contains only one kind of monosaccharide molecule, it is known as a homo-polysaccharide, or homoglycan, whereas those containing more than one kind of monosaccharide are heteropolysaccharides. The most common constituent of polysaccharides is d-glucose, but d-fructose, d-galactose, l-galactose, d-mannose, l-arabinose, and d-xylose are also frequent. Some mono-saccharide derivatives found in polysaccharides include the amino sugars (d-glucosamine and d-galactosamine ) as well as their derivatives (*N*-acetylneuraminic acid and *N*-acetylmuramic acid), and simple sugar acids (glucuronic and iduronic acids). Homopolysaccharides are often named for the sugar unit they contain, so glucose homopolysaccharides are called glucans, while mannose homopolysaccharides are mannans. Polysaccharides differ not only in the nature of their component monosaccharides but also in the length of their chains and in the amount of chain branching that occurs. Although a given sugar residue has only one anomeric carbon and thus can form only one glycosidic linkage with hydroxyl groups on other molecules, each sugar residue carries several hydroxyls, one or more of which may be an acceptor of glycosyl substituents. This ability to form branched structures distinguishes polysaccharides from proteins and nucleic acids, which occur only as linear polymers. 

The main functions played by polysaccharides in Nature are either storage or structural functions. By far the most common storage polysaccharide in plants is starch, which exists in two forms: α-amylose and amylopectin. Structural polysaccharides exhibit properties that are dramatically different from those of the storage polysaccharides, even though the compositions of these two classes are similar. The structural polysaccharide cellulose is the most abundant natural polymer in the world. Found in the cell walls of nearly all plants, included marine algae, cellulose is one of the principal components, providing physical structure and strength. Marine derived cellulose of animal origin, tunicin, extracted from tunicates – invertebrate sea animals – is a material of choice, since it is highly crystalline [1,2,3,4]. 

Polysaccharides of algal origin include alginates, agar and carrageenans. Agar (or agar-agar) is an unbranched polysaccharide obtained from the cell membranes of some species of red algae, primarily from the genuses *Gelidium* and *Gracilaria*, or seaweed, largely used as gelatin and thickener in food industry, and as a gel for electrophoresis in microbiology. Chemically, it is constituted by galactose sugar molecules; it is the primary structural support for the algae’s cell walls. Carrageenans are polysaccharides of galactan with alternating 1,3- and 1,4-linked galactose residues, which fill spaces between the cellulosic plant structure of seaweeds; they are used in the food processing industry for their gelling, thickening and stabilising properties.

Exopolysaccharides (EPSs) are high molecular weight carbohydrate polymers that make up a substantial component of the extracellular polymers surrounding most microbial cells in the marine environment. In recent years, there has been a growing interest in isolating new EPS-producing bacteria, particularly from various extreme marine environments [5]. Many new microbial EPSs with novel chemical compositions, properties and structures have been found to have potential applications in fields such as adhesives, textiles, pharmaceuticals and medicine for anti-cancer, food additives, etc. Most EPSs produced by marine bacteria are linear heteropolysaccharides consisting of three or four different monosaccharides arranged in groups of 10 or less to form repeating units [6]. The mono-saccharides may be pentoses, hexoses, amino sugars, or uronic acids. EPSs possess different types of functional groups; most of EPSs are sulfated and high in uronic content, and this confers them a net negative charge and acidic properties at the pH of seawater (pH ~ 8) [7]. 

Chitin is the second most abundant organic compound in nature after cellulose [8]. Chitin is widely distributed in marine invertebrates, insects, fungi, and yeast [9]. However, chitin is not present in higher plants and higher animals. 

Referring to polysaccharides performing structural functions in marine environment, although all of them are abundant in nature, in the present review we will mainly concentrate on alginate, chitin and its derivative chitosan, as they are important not only as abundant resources, but mainly for their attracting biological properties and potential in the biomedical field.

### General principles of chemical modification

Needs for chemical modification concern mainly the improvement of mechanical properties, biocompatibility, solubility, control of biodegradability and manufacturing and shaping. In particular, modification of alginate and chitin/chitosan can follow different approaches:

- Blending or chemical linkages with synthetic biopolymers;
- Surface coating of micro- or nano-spheres with biocompatible synthetic polymers;
- Crosslinking with different physical or chemical reagents;
- Hydrophobization through alkylation reactions;
- Modulation of guluronic/mannuronic ratio, or of deacetylation degree, respectively.

## 2. Polysaccharides as biomaterials

### Alginate

Over the last few years, medical and pharmaceutical industries have shown an increased interest in biopolymers in general and in alginates in particular. The reason for this increased interest is their usefulness in specific applications, as it enhances efficient treatment of esophageal reflux, creates multiquality calcium fibers for dermatology and wound healing. They are also used for high- and low-gel strength dental impression materials. Besides this, alginate is an effective natural disintegrant, tablet binder and offers an attractive alternative for sustained-release systems. It offers advantages over synthetic polymers as it forms hydrogels under relatively mild pH and temperature and is generally regarded as non-toxic, biocompatible, biodegradable, less expensive and abundantly available in nature; in addition, alginate meets the important requirement of being amenable to sterilization and storage. All these advantages make alginates very useful materials for biomedical applications, especially for controlled delivery of drugs and other biologically active compounds and for the encapsulation of cells. Calcium alginate is a natural haemostat, so alginate based dressings are indicated for bleeding wounds [10]. The gel forming property of alginate helps in removing the dressing without much trauma.

The biopolymer alginate exhibits, like pectin and others, the effect of ionotropic gelation if multivalent cations diffuse directed from one side into the sol. During this sol-gel-transition channel-like pores are created. This effect was already described in the 60s [11]. The dimensions of these pores can be influenced by the chemical conditions, for example concentration of the sol or gelling agent, nature and conformation of the alginate and pH or temperature. Therefore, the phenomenon has lately been explained to be a chemically fixed dissipative structure [12]. The channel-like pores still were obtained if ceramic powder was mixed into the sol to produce ceramic filter membranes consisting of hydroxyapatite or alumina, too [13]. For tissue engineering of bone, the phenomenon of channel-pore structure developed upon cross-linking was introduced as biomaterials by exchanging the toxic copper ions as gelling agent for calcium ions and studying the composite hydrogels [14]. Additionally, nano-crystalline hydroxyapatite could be synchronously precipitated during the sol-gel-transition by adding phosphate ions into the alginate sol [14]. For the reason of higher stability, the content of hydroxyapatite was raised by adding powder in the ratio found inside the bone: one part biopolymer and two parts ceramic phase [15]. Short term softening in relevant media like water, simulated body fluid or DMEM was studied on freeze dried scaffolds and biocompatibility was confirmed by fluorescence microscopy at day 4 of a cell culture experiment with human mesenchymal stem cells (hMSC) even so the scaffolds were stable for three weeks [15]. The proliferation and differentiation of hMSC grown for four weeks has been evaluated on freeze-dried scaffolds with larger pores [15]. The cell number increased four-fold and the osteogenic differentiation marker specific alkaline phosphatase (ALP) activity multiplied three times [16]. 

To contribute for the repair of osteochondral defects of joints, which affects bone and covering cartilage, biphasic but monolithic alginate scaffolds were produced which divide into a HAP containing part and an alginate/hyaluronic acid composite hydrogel [17]. Even consisting of two parts, the channel-like pores run through the whole scaffold crossing the interface, therefore, fusing of two different scaffolds which might cause stability problems at the interface. This problem was solved by producing monolithic scaffolds. 

### Chitin and chitosan

Chitin is almost solely used as a raw material for the production of chitosan and other derivatives. Some wound-covering materials have been developed from chitin non-woven fabrics and threads. Chitin is also used as an excipient and drug carrier in film, gel or powder form for applications involving mucoadhesivity. Another interesting application is in association with chitosan for a hydroxyapatite composite bone-filling material, which forms a self-hardening paste for guided tissue regeneration in treatment of periodontal bony defects [18].

Chitosan is widely employed in many biomedical fields [19,20,21,22,23]. Like alginate, chitosan has the characteristic of forming gels in addition to possessing viscosity-related properties, complete bio-degradability, and even anti-tumor influence [24]. Its bacteriostatic and fungistatic properties are particularly useful for wound treatment. Furthermore, chitosan possesses bioadhesive properties which make it of interest in bioadhesive sustained release formulation required [25,26]. Many chitosan derivatives are also biocompatible and non-toxic with living tissues. Recent studies further indicated that chitosan and its derivatives also are novel scaffold materials for tissue engineering and are-promising non-viral vectors for gene delivery [27,28]. For bone regeneration, several injectable materials basing on chitosan and its derivatives have been used. Chitosan-calcium phosphate (CP) composites appear to have a promising clinical application. Chemically modificated HA (hyaluronic acid)-chitin and chitosan-HA material were reported to be osteoinductive and exhibited rapid degradation and neovascularization *in vivo* [29,30]. Chitosan scaffolds are potentially a useful alternative to synthetic cell scaffolds also for cartilage tissue engineering [31]. Recently, biomineralized alginate/chitosan microcapsules have been proposed as multifunctional scaffolds and delivery vehicles in tissue regeneration of hard and soft tissues [32].

## 3. Alginate: Structure and chemical modification

Alginate is a high-molecular mass polysaccharide extracted from various species of kelp [33]. Alginates are also produced extracellularly by *Pseudomonas aeruginosa* and *Azetobacter vinilandii* [34]. Edward Stanford discovered alginate in 1883 and commercial production, started in 1927, has now expanded to about 50,000 tonnes per year worldwide; 30% of this tonnage is devoted to the food industry, the rest being used in industrial, pharmaceutical and dental applications [35]. The function of alginates in algae is primarily skeletal, with the gel located in the cell wall and intercellular matrix conferring the strength and flexibility necessary to withstand the force of water in which the seaweed grows [36]. 

Alginate is a linear, anionic block copolymer heteropolysaccharide consisting of β-d-mannuronic acid (M) and α-l-guluronic acid (G). The relative amount and sequential distribution of homogeneous M-M segments (M-blocks), homogeneous G-G segments (G-blocks) and alternating M-G segments (MG-blocks), which represent the primary structure of alginate, depend on the producing species, and for marine sources, on seasonal and geographical variations. The primary structure is generally defined by the F_G_ value, which is the fraction of overall guluronic acid residues in the polymer, and by N_G_, the number-average of guluronic units in G-blocks. The alginate is known to form a physical gel by hydrogen bonding at low pH (acid gel), and by ionic interactions with divalent (Ca, Sr, and Ba) or trivalent (Fe(III) and Al) ions, that act as crosslinkers between adjacent polymer chains. G-blocks are the ones mainly responsible of such ionic interactions, as in the presence of multivalent cations they can associate to form aggregates of the “egg-box” type. The term ‘‘Egg Box’’ arises from a similitude model in which the cation fits into electronegative cavities like eggs in an egg-box [37]. Hence, an alginate with a higher level of G sequences presents a higher affinity for cross-linking agents than low G-containing alginates [38]. The selective binding of divalent metal ions and the corresponding gel strength were found to increase in the order: MM block<MG block<GG block [39]. The solution viscosity, molecular weight and primary structure are fundamental to determine the swelling and gelling properties of alginate; the solubility of alginate is also affected by primary structure, ionic strength and pH. Physical properties of alginate gels varies widely, depending on their composition [40], i.e., proportion of G and M residues, the sequential order of these residues, overall molecular weight of the polymer, and calcium ion concentration at the time of gelation. It has been reported that alginates containing a high G content develop a stiffer, more brittle, and more porous gel, which maintains its integrity for long periods of time. During calcium cross-linking, they do not undergo excessive swelling and subsequent shrinking; thus, they can maintain their form in a better manner. Also it has been found that the greater the G content of the gel, the greater is the restriction to solute transport [41]. Conversely, alginates rich in M residues develop softer and less porous beads, which tend to disintegrate easier with time. Alginate with high M content also undergoes a high degree of swelling during calcium crosslinking [37]. Gels with a uniform concentration of alginate can be obtained by cross-linking via the internal setting method [42,43]. This technique allows a controlled gelation of alginate through a slow release of calcium ions, thus leading to the formation of a very regular gel network. In general, this method uses an inactive form of the crosslinking ion, either bound by a sequestering agent such as phosphate, citrate or EDTA, or as a very low solubility salt, as CaSO_4_, or as a salt insoluble at neutral pH, for example CaCO_3_, in association with a slowly hydrolyzing lactone, usually d-glucono-δ-lactone (GDL). Since GDL generates an acidic pH, the calcium ions are gradually released and captured by guluronic residues of alginate. Uniformity and well-controlled material properties are, indeed, necessary in biomedical applications, such as tissue engineering [44,45]. 

It is worth note that physical gels can gradually loose their mechanical stability in biological fluids, due to an outwards flux of crosslinking calcium ions in the surrounding medium. As a consequence, chemical strategies to introduce stable covalent crosslinks using bifunctional crosslinkers, such as glutaraldehyde, have also been developed [46]. Calcium alginate gels are readily destabilized in the presence of calcium complexants EDTA-sodium citrate [47] or monovalent cations [48] and complex anions such as phosphate, citrate, and lactate, which have high affinity for calcium ions. The instability is also caused by the presence of high concentration of non-gelling ions such as sodium and magnesium. Stabilization can be achieved by adding free calcium ions to the medium while maintaining a Na:Ca ratio of less than 25:1 for high-guluronate alginates and 3:1 for low-guluronate alginates. Stabilization of calcium alginate gels by adding other multivalent ions such as Ti and Al has also been reported [49,50]. Another important property of alginate gel is that sol-gel transition occurs without any alteration of temperature. The gel can be easily converted into a solution by adding sodium, magnesium, and EDTA. 

### Chemical modification

**Scheme 1:**
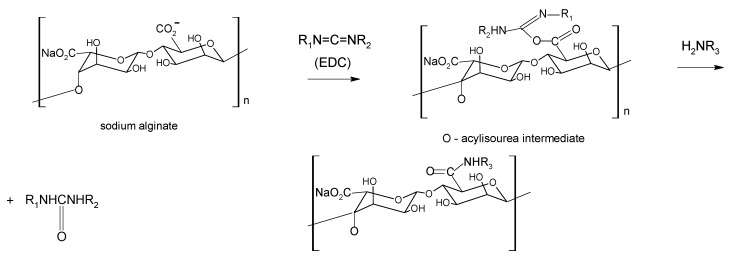


Aqueous carbodiimide chemistry, using 1-ethyl-(dimethylaminopropyl)carbodiimide (EDC) as water soluble carbodiimide, is widely used to couple carboxylic groups on alginate with molecules containing primary or secondary amines [51,52] as well as dihydrazides [53] (Scheme 1). A co-reactant as *N*-hydroxysulfosuccinimide (sulfo-NHS) is often used to stabilize the reactive EDC-intermediates against hydrolysis, raising the efficiency of amide bond formation [54].

**Scheme 2:**
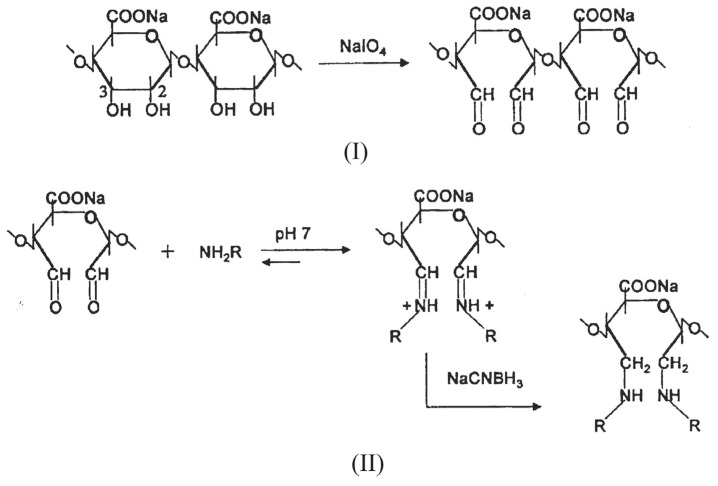


Such a reaction, however, consumes the carboxyl groups, essential for the gelation process. To overcome this inconvenient, the introduction of aldehydic groups, more reactive than hydroxyl or carboxylic ones, onto sodium alginate via periodate oxidation represent a selected approach to activate the polysaccharide for the successive chemical modifications [55,56,57]. Periodate oxidation selectively cleaves the vicinal glycols in polysaccharides to form their dihaldeyde derivatives (Scheme 2, I.) The reaction proceeds with significant depolymerization of alginate. As the depolymerization is a free-radical mediated reaction due to oxidation of impurities present, the addition of aliphatic alcohols, usually isopropanol, that act as radical scavenger prevents the depolymerization giving rise to oxidized alginate of higher Mw [58,59]. Depolymerization also depends on primary structure; as a matter of fact, the oxidation is still more degradative when the content of mannuronic and guluronic alternating blocks (MG-blocks) is high, as chain scission preferentially takes place at atipical sugar units. 

The subsequent step involves the condensation of the aldehydic groups with amines via a reductive amination reaction. (Scheme 2, II). The most frequently employed reducing agents are sodium borohydride (NaBH_4_) and sodium cianoborohydrurecyanoborohydride (NaCNBH_3_). The lastlatter has the advantage to giveof affording a rapid reduction of the intermediates imine groups at pH 6-7, while the competitive reduction of carboxylic groups to the corresponding alcohols is negligible in this pH range.

The reaction is often used for hydrophobization of alginate by reaction with medium-long chain (C_8 _- C_16_) alkylic amines. Hydrophobized derivatives of alginate demonstrate amphiphilic properties in aqueous medium and have been widely investigated for a variety of applications, such as a material for immobilizing enzymes. Moreover, hydrophobization of alginate by insertion of alkylic chains is commonly reported to promote protein absorption and, consequently, cell anchorage. For several applications, as additive in food and cosmetics, the gelling ability of alginate may be inhibited by chemical modification with propylene oxide.

**Scheme 3:**
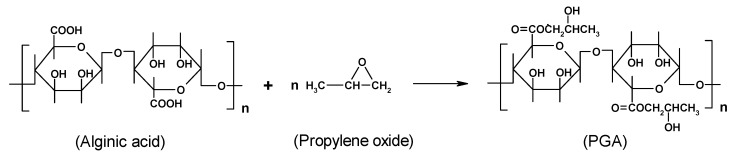


PGA (propylene glycol alginate) is the only, commercially available, chemically modified alginate (coded as E405). PGA is made by contacting a partially neutralised alginic acid with propylene oxide gas under pressure (Scheme 3). The propylene oxide reacts exothermically with the alginic acid to form a mixed primary/secondary ester. The partial or total substitution of acid groups with hydroxyester brings about a reduced or absent capacity of gelling, and thus the alginate can also be used as densifier in acidic solutions. 

## 4. Alginate-based materials for drug-delivery applications

Alginate has been successfully used as a matrix for the entrapment and/or delivery of biological agents, such as drugs and proteins. In particular proteins can be loaded and released by alginate matrices without loss of their biological activity because of the relatively mild gelation process of alginate. In pharmaceutical formulations, the alginate gel can be prepared prior to use, or it can spontaneously form *in situ* in physiological fluids, by low pH and/or calcium ions naturally present in the site of administration. Alternatively, the gelling agent can be added either as a part of the formulation or separately administered. The microencapsulation technique has been specifically developed for the oral delivery of proteins, as they are quickly denaturated and degraded in the hostile environment of the stomach. The protein is encapsulated in a core material that, in turn, is coated with a biocompatible, semi permeable membrane, which controls the release rate of the protein while protecting it from biodegradation. Several examples are reported, in which alginate is used in combination with polyethyleneglycol (PEG). Alginate gels can act as core materials in this application, while PEG, which exhibits certain useful properties such as protein resistance, low toxicity and immunogenicity, together with the ability to preserve the biological properties of proteins [60,61], can act as a coating membrane. A chitosan/PEG-alginate microencapsulation process [62], applie to biological macro-molecules such as albumin or hirudin, was reported to be a good candidate for oral delivery of bioactive peptides [63]. 

In general, drugs with non-favourable solid state properties, such as low solubility, benefit from encapsulation in an amorphous gel matrix. Recently, the synthesis of alginate bearing cyclodextrin (CD) molecules covalently linked on polymer chains for a sustained release of hydrophobic drugs has been reported [53,64]. Such CD-derivatives of alginate are promising as they exhibit cumulative properties of size specificity of CD and transport properties of polymer matrix. Solid preparations based on alginate such as oral tablets [65], microcapsules [66], implants [67], topical delivery systems [68,69] are currently disposable. The oral route is considered the preferred administration route. Tablets are the most abundant dosage form, due to their convenience, easy of preparation and handling. The simpler tablet formulations are prepared by direct compression of a mechanical mixture of the various ingredients, without any need of granulation or coating. Tablets based on alginate have been prepared by direct compression, as well as wet or dry granulation and coating with various techniques [65,70,71]. In monolithic tablets made from alginate (in which the drug is homogeneously dispersed), drug release is controlled by the formation of a viscous hydrated layer around the tablet, in which water penetrates, that acts as a diffusional barrier. Water soluble drugs are mainly released by diffusion across this gel layer, while poorly soluble drugs are mainly released by erosion of the tablet.

Micro- and nanocapsules can be prepared from alginate. Microcapsules (typically > 200 μm) are simply obtained by dropping an aqueous solution of alginate into a gelling solution, either acid (pH< 4) or, more usually, containing calcium chloride (CaCl_2_) as cross-linking agent. Microspheres of lower dimension (< 10 μm) are produced by a water-in-oil emulsification process using an ultrasonicator [72]. To obtain a stable water-in-oil emulsion a surfactant agent is used. An aqueous CaCl_2_ solution is then added to the emulsion under stirring to allow ionotropic gelation of the particles. 

The main shortcomings of alginate devices are their rapid erosion at neutral pH and low adhesion to mucosal tissues, which is further reduced upon crosslinking. Bioadhesive formulations [71,73,74], or formulations with prolonged gastric residence times [75] made from alginate have been reported. In these works, alginate was used in combination with chitosan, polylysine or vegetable oils. More recently, alginate beads for floating drug delivery systems (FDDS) have been prepared [76]. FDDS have a lower density than gastric fluids, so their gastric residence time is longer. Floating alginate beads are easily obtained by dropping an alginate solution containing a foaming agent such as CaCO_3_ or NaHCO_3_ in CaCl_2_/acetic acid. The CO_2_ gas produced remains entrapped inside the beads, which show low density and high porosity. Modification of polysaccharides by introducing acrylic polymer chains is used to obtain a finer control over drug release rate and to improve adhesion to biological substrates [77,78,79,80,81,82,83]. Hydrogels based on crosslinked poly(acrylic acid) [84,85] have been reported to adhere to mucus providing a barrier against irritations and inflammations of membranes of the gastrointestinal system. Acrylic polymers containing amine functionality, as poly(dimethylamino-ethylacrylate, DMAEA), in combination with glycolic residues have been demonstrated to show good bioadhesion and mucoadhesion [86]. 

### Novel alginate-acrylic polymers as a platform for drug delivery

**Figure 1:**
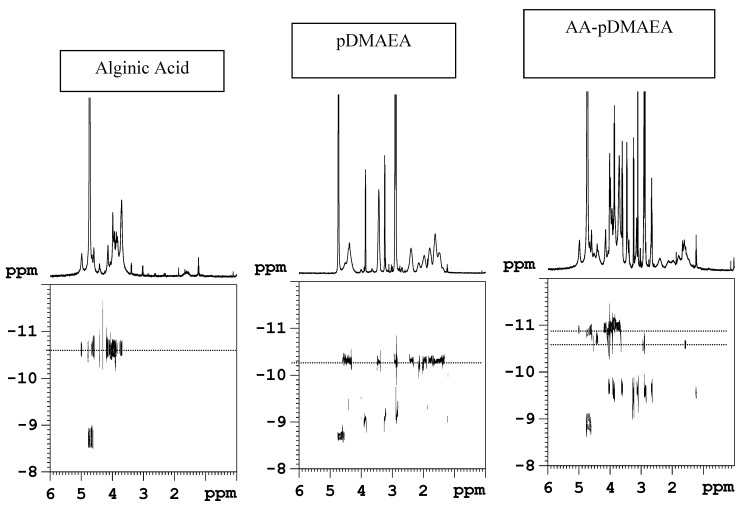
^1^H-detected DOSY maps in phosphate buffered (pD=7) D_2_O solution 0.1 M NaCl at 27°C. The ^1^H spectra are shown as horizontal projections.

Following the above reported examples, the chemical modification of alginate with both poly(acrylic acid) (AA-pAcrAc) and polyDMAEA (AA-pDMAEA) through radical grafting of acrylic monomers has been reported, with the aim to modulate the time of erosion, the rate of release of drugs and the adhesion to substrates [87]. Acrylamide and acrylic acid modified chitosan obtained by a redox- type initiation are described. [77,78,82]. The radical polymerization of an acrylic monomer initiated by peroxide is normally performed directly in the presence of the polysaccharide. In our approach, an original two-step procedure was used to minimize secondary reactions such as, for example, the free homopolymerization reaction of the acrylic monomer; besides, the high viscosity of alginate solution might hinder the polymerization because of the slow diffusion rate of monomers and growing chains, with consequent low conversion. To overcome these problems, as a first step acrylic monomers have been pre-polymerized at 100°C, to promote the disproportionation reaction as the prevalent termination process. It is in fact reported that at this temperature the bimolecular reaction between two acrylic polymeric radicals prevails, that brings to the transfer of a hydrogen atom with formation of a double bond at the end of the acrylic chain [88]. In the second step, coupling of such vinyl-terminated acrylic chains with sodium alginate in presence of potassium persulphate was performed. As the literature on acrylic modified polysaccharides does not clearly indicate the mechanism of grafting (the reported data refer either to a template type polymerization [79,80], or to a true chemical grafting process [89,90]), an investigation aimed at elucidating the chemical structure (graft copolymer or inter-polymer complex) of the obtained polymers via diffusion-ordered NMR spectroscopy (DOSY) [91] was performed. This original technique is an innovative convenient way of displaying the molecular self-diffusion information in a bi-dimensional array, with the NMR spectrum in one dimension and the self-diffusion coefficient in the other one. DOSY has been successfully used for the analysis of mixtures [92], for the characterization of aggregates [93], for the molecular weight determination of uncharged polysaccharides [94]. It is believed that DOSY is an appropriate technique also to distinguish between copolymers and interpolymer complexes. The DOSY maps obtained in the case of AA-pDMAEA sample, together with those of plain alginate and plain pDMAEA as comparison, are shown in Figure 1. It is evident that the two components retain different values of the diffusion coefficient in the AA-pDMAEA sample. As a consequence, we may safely assess that no covalent neither ionic bond exists between the two components of the mixture, namely alginate and polyDMAEA. Analogous results were obtained for acrylic acid-grafted alginate (AA-pAcrAc). Moreover, in the DOSY map it is also possible to evidence the presence of low molecular weight compounds showing a fast value of the diffusion coefficient. This is not unexpected, as peroxides may cause a degradation of AA as side reaction [95], and the formation of oligomers during the radical polymerization of acrylics is also well known. In conclusion, we may hypothesize in both cases the formation of a stable interpolymer complex based on non-ionic inter-chain interactions between the two polymers.

**Figure 2:**
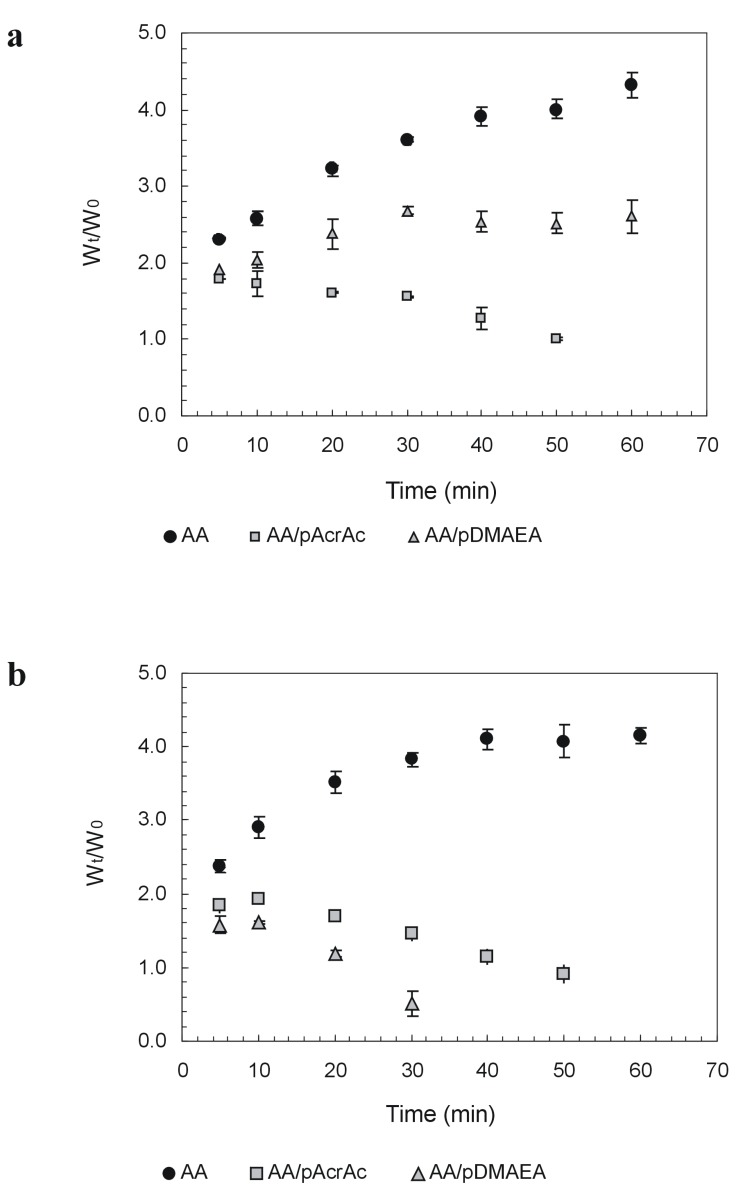
Weight change of alginate tablets in SGF at pH 1.2 (a) and SIF at pH 6.8 (b). Data are the mean of three replicates.

The influence of acrylic polymers on swelling and dissolution rate characteristics of alginate tablets prepared by direct compression has been evaluated, as it will determine differences in drug release properties. Tablet weight change in aqueous media was evaluated in Simulated Gastric Fluid (SGF, pH 1.2) and Simulated Intestinal Fluid (SIF, pH 6.8) to simulate *in vivo* conditions. In general, an initial increase of weight due to water uptake (swelling) is detected, followed by a weight decrease due to erosion of the tablet. The results, reported in Figure 2, show that modification with basic moieties decreases polymer hydrophilicity, particularly at pH 1.2. This may be due to a partial shielding of carboxylic functions that interact with protonated amines. No erosion is found within 60 minutes for alginate and AA-pDMAEA. On the other hand, at pH 6.8 the decrease in the amount of positive charges in the network disturbs the intermolecular bonding between alginate chains and results in a faster onset of tablet erosion (after about 10 min.). Very interestingly, the increase in the amount of acidic functions in the alginate network in AA-pAcrAc samples contributed to speed up the induction time for tablet erosion independently of the pH of the medium. 

**Figure 3:**
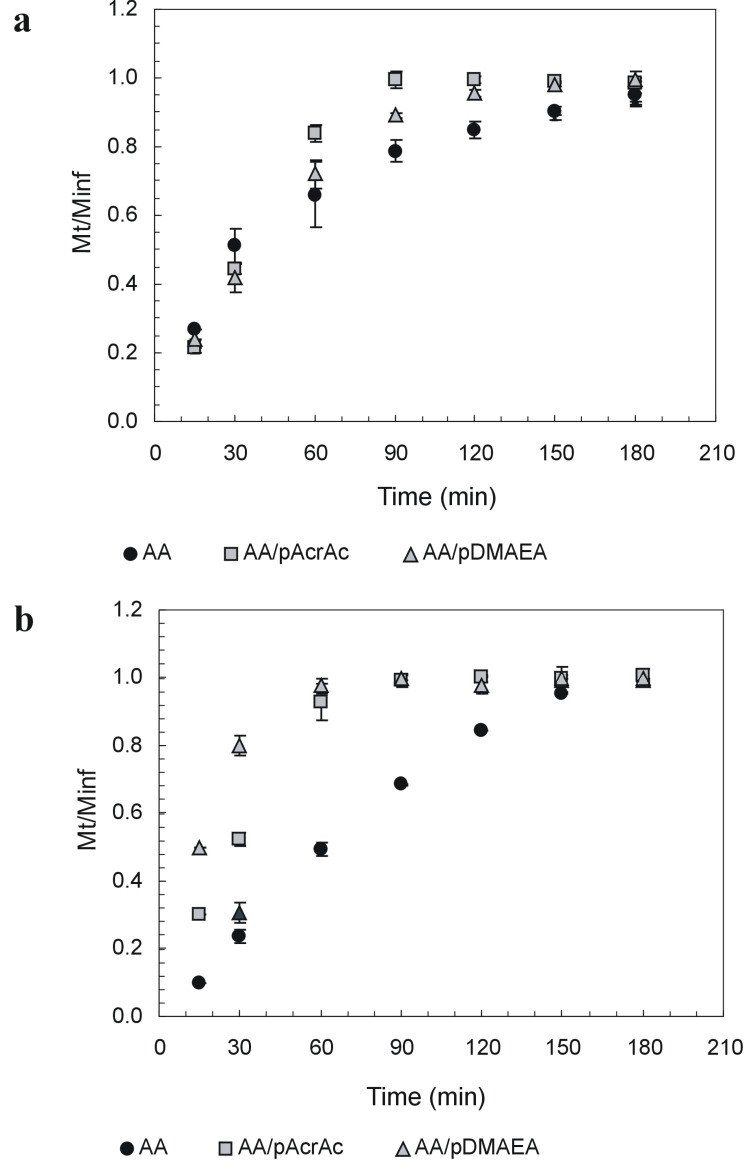
Release of 7-(β-hydroxyethyl)theophyllinefrom alginate tablets in SGF at pH 1.2 (a) and SIF at pH 6.8 (b). Data are the mean of three replicates.

Drug release studies were performed using a freely soluble, unionized drug molecule, namely 7-(β-hydroxyethyl)theophylline (ETO), as release from alginate matrices is strongly affected by the formation of intermolecular bonds between positively-charged drug molecules and AA network [96]. Release profiles in the two pH conditions are reported in Figure 3. The release from modified alginates tablets was always faster than from pure AA, particularly at pH 6.8, where ETO release from AA-pAcrAc and AA-pDMAEA tablets was completed in about 60 minutes. To investigate on the mechanism release (diffusion across the hydrated outer layer and/or tablet erosion), the extent of matrix erosion when all the drug had been released from the tablets was evaluated too (Table 1). The erosion extent was evaluated by weighing the residual tablet after drying until constant weight (40°C under vacuum). 

As it can be seen, AA-pAcrAc tablets were completely degraded at both pH values within 90 min, whereas AA-pDMAEA tablets eroded at a rate depending on medium pH. The erosion of AA-pDMAEA tablets was very fast at pH 6.8, but much slower at pH 1.2. These results suggest that ETO release from AA-pDMAEA tablets at acidic pH is governed by both diffusion through the hydrated outer layer and polymer dissolution, while at pH 6.8, it is mainly controlled by polymer erosion rate. We have so demonstrated a dependence of release rate and mechanism from acrylic polymer type and medium pH. 

**Table 1:** Extent of tablet erosion when ETO release is complete.

*Sample*	*pH 1.2*		*pH 6.8*	
	*T_1.0_*	*Weight loss*	*T_1.0_*	*Weight loss*
	*(min) ^a^*	*%*	*(min) ^a^*	*%*
*AA*	*180*	*32±2*	*150*	*90±3*
*AA-pAcrAc*	*90*	*100±2*	*60*	*100±5*
*AA-pDMAEA*	*150*	*78±2*	*60*	*100±4*

^a^ Time at which ETO release is over.

The effect of alginate modification on adhesion properties was also investigated. Measurements were performed using a Instron Model 4301 dynamometer testing system. Samples affixed to the moving crosshead were put in contact with a wet glass surface as adhesion substrate clamped onto the stationary platform; after 10 minutes contact time, the crosshead was raised at a constant rate up to complete detachment between the sample and the glass substrate. 

Data were reported as the maximum force required for complete detachment per cross sectional area (Table 2). Although the adhesion between the tablet and the glass is stronger than that between the tablet and the mucosa [81], the parameters obtained in this way can be taken into consideration to evidence differences between alginate and modified alginate. The differences are, indeed, noticeable, as the work of detachment is about the triple in AA-pAcrAc and AA-pDMAEA with respect to that of the pure alginate. We believe that the introduction of pAcrAc or pDMAEA chains may improve chain mobility and, in turn, increase the number of interconnections between the tablet and the substrate, leading to better adhesion. This behavior is welcome for any application in which the systems are used as drug delivery in contact with mucosae. 

**Table 2:** Adhesion parameters.

Sample	Work of Detachment
	(J/m^2^)
AA	9.4
AA-pAcrAc	29.4
AA-pDMAEA	29.6

## 5. Alginate for cell immobilization

Among the possible applications of alginate gel systems, one of the most promising is for cell immobilization. Gel-entrapments allow suspension cells to be cultivated in several types of bioreactors to achieve high cell densities. In cell immobilization applications, the main drawback of alginate matrix gels is represented by their high density of network, which limits the cell growth [51]; moreover, cell anchorage, a strict requirement for survival, is limited on alginate gels, because of its hydrophilic nature. PEG copolymers are used to improve the biocompatibility of polysaccharides. Several PEG-alginate systems for cell entrapment have been reported [97]; not many examples of PEG-alginate copolymers are found in literature. 

### Synthesis of a novel alginate-poly(ethylene glycol) graft copolymer

**Scheme 4:**
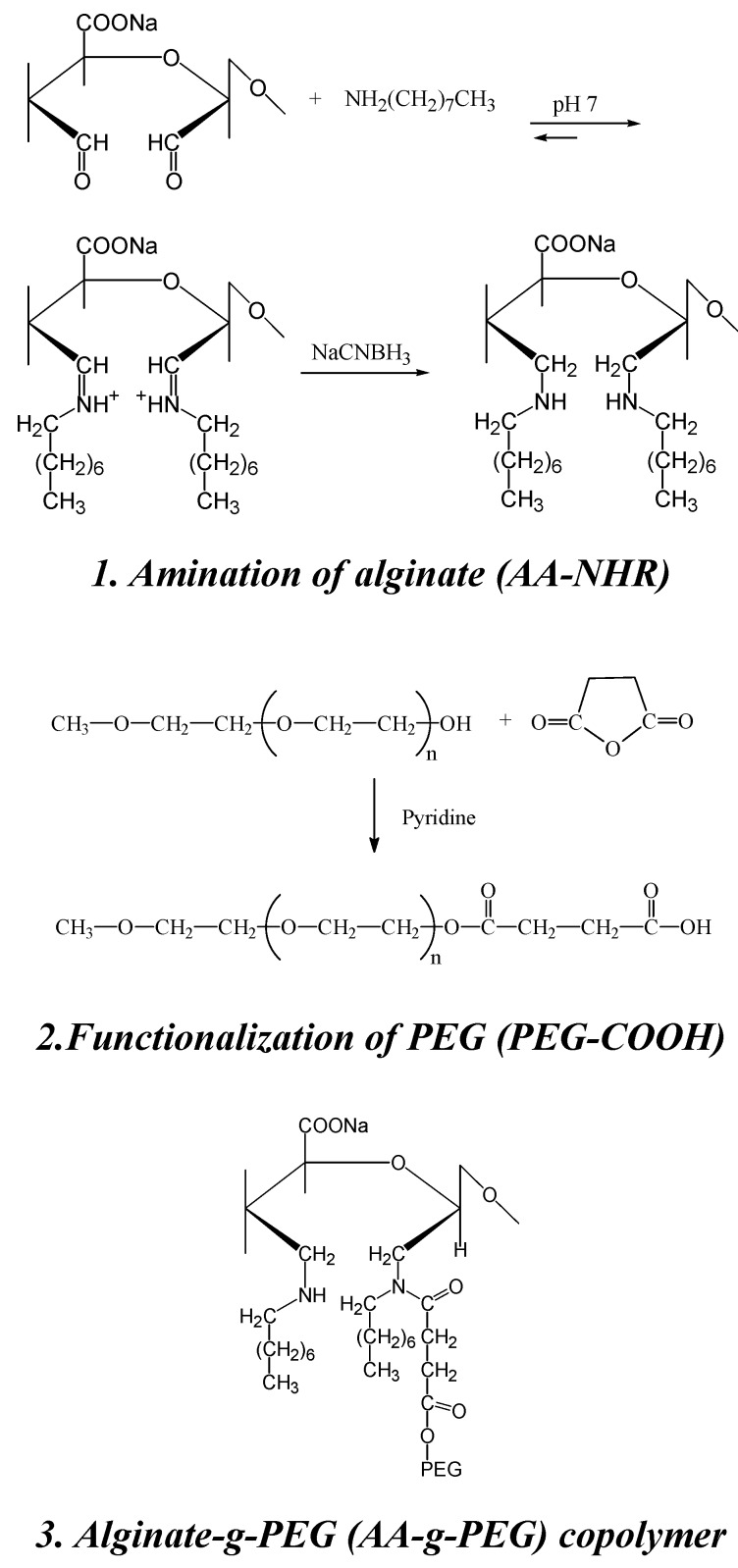


Recently, a protocol for the synthesis of new alginate-g-PEG copolymers that retain the gelation characteristics of alginate has been described [98]. The reaction proceeds through well known synthesis routes and does not involve the carboxylic groups of alginate, essential for the gelation process. Amines are introduced onto alginate to allow the coupling, via carbodiimide chemistry, with a PEG mono-terminated with carboxylic acid groups. The introduction of secondary amines was performed in two successive steps: oxidation of alginate with sodium metaperiodate, to generate aldehydic groups, followed by reductive amination of aldehydic groups with octyl amine. PEG mono-methyl ether (mPEG, Mw. 2000) was functionalized by reaction with succinic anhydride using pyridine as catalyst, to give an acid end-functionalized PEG (PEG-COOH) (Scheme 4).

**Figure 5:**
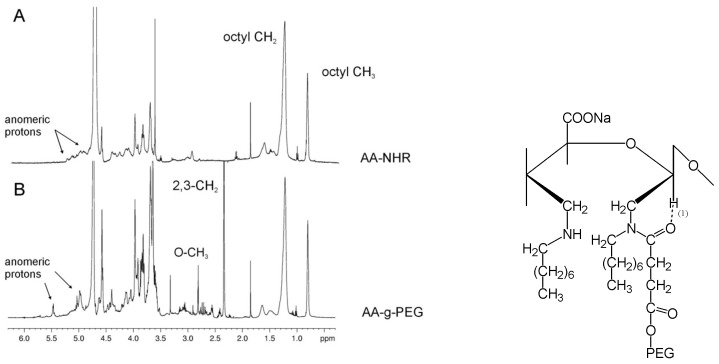
600-MHz proton spectra of AA-NHR and AA-g-PEG*.

The occurrence of a chemical bond was confirmed using one-dimensional ^1^H-NMR spectroscopy. A comparison between the ^1^H-NMR spectra of AA-NHR and AA-g-PEG (Figure 5A, B) provides clear evidence of the presence of a chemical bond between PEG and the modified alginate. The two proton peaks resonating at 3.36 (terminal O-CH_3_) and 2.66 ppm (CH_2_ at positions 2 and 3) in the PEG, respectively, are observed upfield-shifted at 2.81 and 2.34 ppm in AA-g-PEG with respect to PEG-COOH. Furthermore, the methyl and methylene protons of the octyl chain are also upfield-shifted at 0.80 and 1.22 ppm, respectively. The anomeric proton region is also affected by the addition of PEG to alginate. In the AA-NHR the anomeric protons are all clustered in the region 5.3-4.9 ppm (Figure 5A), but upon addition of PEG they group in two well-separated regions at 5.47 and 4.97 ppm (Figure 5B). We attribute such a spectral change to the formation of a hydrogen bond between the anomeric proton of the open alginate rings and the amidic carbonyl of the attached PEG, forming a stable six-membered ring as sketched in the Figure. The presence of grafted PEG molecules inside alginate gels is thought to increase the pores dimension and induce improved cell anchorage. Gelation ability of alginate is not significantly affected by the presence of grafted PEG molecules.

* Reprinted with permission from Laurienzo *et al.* Synthesis of a novel alginate-poly(ethylene glycol) graft copolymer for cell immobilization. *Carbohydr. Polym.* **2005***, 62*, pp. 277, 278. Copyright Elsevier 2007.

## 6. Chitin and chitosan: structure and chemical modification

Chitin is a semi-crystalline homopolymer of β-(1→4)-linked *N*-acetyl-D-glucosamine. Chitin is widely available from a variety of source, among which the principal one is shellfish waste such as shrimps, crabs, and crawfish [99]. It also exists naturally in a few species of fungi. Generally, the shell of selected crustacean was reported by Knorr [100] to consist of 30-40% protein, 30-50% calcium carbonate and calcium phosphate, and 20-30% chitin. Chitin fibrils are found in association with proteins and are embedded in a matrix of calcium carbonate and phosphate that also contains protein. Protein in the matrix is hardened by a tanning process [101]. Studies of Asford and co-workers [102] demonstrated that chitin represents 14-27% and 13-15% of the dry weight of shrimp and crab processing wastes, respectively. In industrial processing, chitin is extracted from crustaceans by acid treatment to dissolve calcium carbonate followed by alkaline extraction to solubilize proteins. The resulting chitin needs to graded in terms of purity and colour for further biomedical applications. Chitin can be used in blends with natural or synthetic polymers; it can be crosslinked by epichlorhydrin and glutaraldehyde. Because of its biodegradability, nontoxicity, antibacterial and gel-forming properties, chitin is widely employed in biomedical field [103,104,105,106]. 

Chitosan is a fiber-like substance derived from chitin. Chitin and chitosan have similar chemical structures. Chitin is made up of a linear chain of acetylglucosamine groups, while chitosan is obtained by removing enough acetyl groups for the molecule to be soluble in most dilute acids. This process is called deacetylation. The actual difference between chitin and chitosan is the acetyl content of the polymer [107]. 

### Definition and Composition of Chitosan: Degree of Deacetylation (DD)

The deacetylation involves the removal of acetyl groups from the molecular chain of chitin, leaving behind a compound (chitosan) with a high degree chemical reactive amino groups (‑NH_2_). This makes the degree of deacetylation (DD) an important property in chitosan production as it affects the physicochemical properties, hence determines its appropriate applications. Deacetylation also affects the biodegradability and immunological activity [108]. A sharp nomenclature border has not been defined between chitin and chitosan based on the degree of *N*-deacetylation. In an earlier study by Rudall [109], he reviewed evidences suggesting that approximately one in every six to seven residues in the chain has a proportion of free amino groups that manifests some histochemical properties. In any case, the degree of deacetylation can be employed to differentiate between chitin and chitosan because it determines the content of free amino groups in the polysaccharides. In fact there are two advantages of chitosan over chitin. In order to dissolve chitin, highly toxic solvents such as lithium chloride and dimethylacetamide are used whereas chitosan is readily dissolved in diluted acetic acid. The second advantage is that chitosan possesses free amine groups which are an active site in many chemical reactions [110]. 

The degree of deacetylation of chitosan ranges from 56% to 99% with an average of 80%, depending on the crustacean species and the preparation methods [111,112]. Chitin with a degree of deacetylation of 75% or above is generally known as chitosan [110]. Various methods have been reported for the determination of the degree of deacetylation of chitosan. These included ninhydrin test, linear potentiometric titration, near-infrared spectroscopy, nuclear magnetic resonance spectroscopy, hydrogen bromide titrometry, infrared spectroscopy, and first derivative UV-spectro-photometry [113]. The IR spectroscopy method, which was first proposed by Moore and Roberts [114], is commonly used for the estimation of chitosan DD values. This method has a number of advantages and disadvantages. First, it is relatively fast and unlike other spectroscopic methods, does not require purity of the sample to be tested nor require dissolution of the chitosan sample in an aqueous solvent. However, the IR method utilizing baseline for DD calculation, and as such there may be possible argument for employment of different baseline which would inevitably contribute to variation in the DD values. Secondly, sample preparation, type of instrument used and conditions may influence the sample analysis. Since chitosan is hygroscopic in nature and samples with lower DD may absorb more moisture than those with higher DD, it is essential that the samples under analysis be completely dry [113]. 

### Characteristics of Chitosan

Chitosan is a non toxic, biodegradable polymer of high molecular weight, and is very much similar to cellulose, a plant fiber. The only difference between chitosan and cellulose is the amine (-NH2) group in the position C-2 of chitosan instead of the hydroxyl (-OH) group found in cellulose. However, unlike plant fiber, chitosan possesses positive ionic charges, which give it the ability to chemically bind with negatively charged fats, lipids, cholesterol, metal ions, proteins, and macromolecules [115]. In this respect, chitin and chitosan have attained increasing commercial interest as suitable resource materials due to their excellent properties including biocompatibility, biodegradability, adsorption, and ability to form films, and to chelate metal ions. 

However, both chitin and chitosan exhibit limitations in their reactivity and processability. The scarce water solubility is the major limiting factor in their application. For a breakthrough in utilization, chemical modification to introduce a variety of functional groups will be a key point because such procedure would not change the fundamental skeleton of polymers and would keep the original physicochemical and biochemical properties, depending on the nature of introduced group and finally would bring new or improved properties.

### Chitin chemical modification

Chemical modifications of chitin are generally difficult owing the lack of solubility as reactions under heterogeneous conditions are accompanied by various problems such as the poor extent of reaction, difficulty in region-selective substitution, structural ambiguity of products and partial degradation due to the severe reaction conditions.

Carboxymethylchitin is one of the most studied chitin derivatives, obtained by adding monochloroacetic acid to chitin previously treated with sodium hydroxide at different concentrations, until a neutral viscous milky solution was obtained. The water soluble product was carboxymethylchitin. For each concentration of alkali used, one set of carboxymethylation degree was allowed to take place [116]. This product activates peritoneal macrophages *in vivo*, suppresses the growth of tumor cells in mice, and stimulates nonspecific host resistance against *Escherichia Coli* infections [117]. Chitin can be used in blends with natural or synthetic polymers; it can be crosslinked by epichlorhydrin and glutaraldehyde [118]. 

The most important chitin derivative is chitosan, obtained by its partial deacetylation under alkaline conditions or by enzymatic hydrolysis in presence of chitin deacetylase. Because of the semicrystalline morphology of chitin, chitosans obtained by a solid-state reaction have a heterogeneous distribution of acetyl groups along the chain. On the other hand, when chitin is treated with concentrated aqueous sodium hydroxide, *N*-deacetylation proceeds smoothly and homogeneously deacetylated samples were obtained [119]. 

### Chitosan chemical modification

Among the many chemical derivatives of chitosan mentioned in literature [120], one can differentiate specific reaction involving the –NH_2_ group at the C-2 position or non-specific reactions of –OH groups at C-3 and C-6 positions (especially esterification and etherification). The more common and easy reactions involving the amino group at C-2 position are the quaternization and the reductive amination with aldehydes. [121]. 

### O-/N- carboxyalkylation

*O*-/*N*-carboxymethylchitosan (CM-chitosan) is one of the most investigated derivatives of chitosan, obtained under controlled reaction conditions with sodium monochloroacetate. This amphoteric polyelectrolyte has attracted considerable interest in a wide range of biomedical applications, such as wound dressings, artificial bone and skin, bacteriostatic agents, and blood anticoagulants, due to its unique chemical, physical, and biological properties, especially its excellent biocompatibility [122,123,124,125,126]. The presence of both carboxyl groups and amino groups in CM-chitosan macromolecules elicits special physicochemical and biophysical properties. It is interesting for pharmaceutical applications because of their novel properties, especially for controlled or sustained drug-delivering systems [127]. *N*-carboxymethylation of chitosan is effected through Schiff base formation from the free amino group of chitosan with an aldehyde or keto group and the successive reduction with cyanoborohydride or sodium borohydride [128]. 

This method results in regioselective carboxymethylation of the amino group, so the product of reaction is a well-defined derivative. Several *N*-carboxyalkylated chitosans were prepared via Schiff base formation from carboxylic acids having aldehyde or keto groups [129,130,131]. The resulting carboxyalkylated derivatives find applications as biomedical materials and fungistatic agents [132,133]. 

Liu *et al*. [134] utilized *O*-carboxymethylchitosan in order to develop a water-soluble matrix polymer for controlled drug release. OCM-chitosan microspheres containing antibiotic drug pazufloxacin mesilate were prepared by the emulsion method and successively crosslinked with glutaraldehyde. 

### Sulfonation

Chemical modification of the amino and hydroxyl groups of chitosan with sulphate can generate products for pharmaceutical applications. Sulfonation reactions of polysaccharides can give rise to a structural heterogeneity in polymer chain, but on the other hand some structures that emerge from random distribution can reveal good features for biological functions. Sulphated chitosans, that represent the nearest structural analogues of the natural blood anticoagulant heparin, show anticoagulant, antisclerotic, antitumor and antiviral activities [135,136,137,138]. Chitosan derivatives having *N*- and/or *O*-sulphate groups either alone or in conjunction with other substituents have been widely examined as potential heparinoids [139]. Vikhoreva *et al*. [140] synthesized chitosan sulphates by sulfation of low molecular weight chitosan (Mw 9000–35,000 Da). They used oleum as sulfating agent and dimethylformamide as medium and demonstrated that chitosan sulphates with reduced molecular weight show a regular increase of anti-coagulant activity, like heparins. Holme *et al*. [141] converted amino groups of chitosan, with low *N*-acetyl content, into anionic centers through *N*-sulfation. They used trimethylamine-sulfur trioxide, which is known to effect selective *N*-sulfation of amino alcohols [142]. Selective *O*-sulfonation of chitosan was performed by Zhang *et al*. [143]. They prepared *N*-alkyl-*O*-sulphate chitosan by treating *N*-octyl-chitosan with DMF and chlorosulphonic acid. The thermal stability of such *N*-alkyl-*O*-sulphated chitosan decreased with respect to that of the original chitosan. The introduction of substituents into polysaccharide structures disrupt the crystalline structure of chitosan, especially due to loss of hydrogen bonding. *N*-Alkyl-*O*-sulphate chitosan has an amphiphilic character due to the presence of hydrophobic moieties, alkyl chains, and hydrophilic moieties, sulphate groups. Because of this, it has the capacity to form micelles in water and can be used as a potential drug carrier. 

### Acylation

A variety of acylation reactions of chitosan are possible by using different acylating agents, such as aliphatic carboxylic acid chlorides (hexanoyl, dodecanoyl and tetradecanoyl chlorides), cyclic anhydrides, cyclic esters. The acylation reaction is not regioselective. *N,O*-acylated chitosans were prepared with acyl chlorides in methanesulfonic acid [144,145]; the derivatives of 4-chlorobutyl and decanoyl chlorides showed higher fungidal activities than chitosan [146]. Selectively *N*-acylated chitosan have been obtained by Lee *et al*. [147,148] with butanoic, hexanoic and benzoic anhydride under homogeneous conditions in the presence of methanol. Such a chemical modification of chitosan was carried out to induce a hydrophobic nature to the hydrophilic chitosan backbone and to prevent particle aggregation. Chitosan nanoparticulate systems for intravenous administration, with an assumption that engineered nanoparticle systems can adsorb overdosed drugs selectively and rapidly, and reduce their free blood concentration to a safe level, were developed [149]. 

**Scheme 5:**
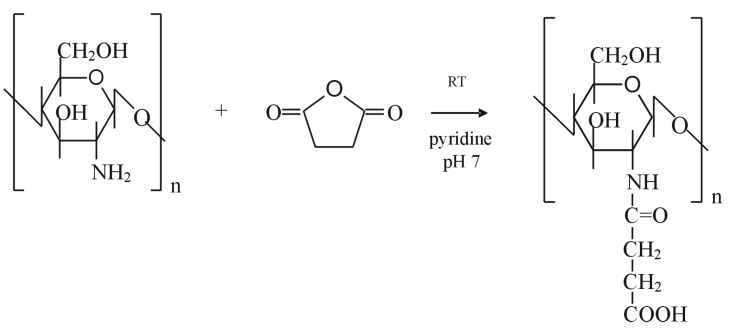


Chitosan can be modified with succinic anhydride in order to obtain a water soluble polymer, N-succinylchitosan. The reaction, described by Aiedeh *et al*. [150], is performed under homogeneous conditions in presence of pyridine and lead to a high degree of chitosan succinylation. The succinylation reaction (illustrated in Scheme 5) consists of a condensation reaction between the polysaccharide amine group and the electrophilic carbonyl group of the anhydride. The reaction involves the formation of an amidic bond with opening of the anhydride ring:

### Sugar-modified chitosan

**Scheme 6:**
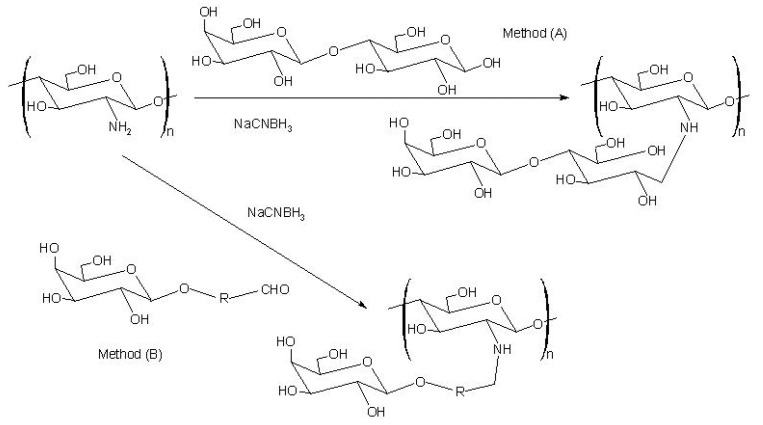


The first report on the modification of chitosan with sugars was by Hall and Yalpani [151,152]. They synthesized sugar-bound chitosan by reductive *N*-alkylation with NaCNBH_3_ using either an unmodified disaccharide (Scheme 6: method A) or a monosaccharide-aldehyde derivative (Scheme 6: method B). This type of modification has generally been used to introduce cell-specific sugars onto chitosan. 

Following this route, Kato *et al*. prepared lactosaminated *N*-succinyl-chitosan and its fluorescein thiocarbanyl derivative as a liver-specific drug carrier in mice through a sialoglycoprotein receptor [153]. Galactosylated chitosan prepared from lactobionic acid (LA) and chitosan with 1-ethyl-3-(3-dimethylaminopropyl)-carbodiimide (EDC) and N-hydroxysuccinimide (NHS) showed promising as a synthetic extracellular matrix for hepatocyte attachment [154]. 

Yang *et al*. [155,156] prepared the chitosan derivatives through the reductive *N*-alkylation of chitosan as described by Sashiwa and Shigemasa [157] with various mono- and disaccharides. *N*-Alkylated chitosan effectively showed solubility at neutral and basic pH region. Moreover, some derivatives substituted with disaccharides including lactose, maltose and cellobiose showed the solubility at all pH range. These derivatives will overcome the application limit of chitosan represented by its reduced solubility. 

### Graft copolymerization

The possibility of grafting synthetic polymer to chitosan has attracted big attention in the last years as a new way to modify the polysaccharide and develop practically useful derivatives. Graft copolymerization reactions introduce side chains and lead to the formation of novel types of tailored hybrid materials composed of natural and synthetic polymers. Grafting chitosan is a common way to improve chitosan properties such as formation of inclusion complexes [158], bacteriostatic effect [159], or to enhance adsorption properties [160,161]. Although the grafting of chitosan modifies its properties, it is possible to retain some interesting characteristics such as mucoadhesivity [162], biocompatibility [163,164] and biodegradability [165].

Many routes for chitosan grafting have been investigated, such as ring opening polymerization of lactides. Copolymers based on lactic acid have been widely used in sutures and pH-sensitive drug release systems because of their biodegradability. Luckachan *et al*. [166] developed chitosan/oligo(l-lactide) graft copolymers by ring opening polymerisation of l-lactide in DMSO at 90°C under a nitrogen atmosphere using Ti(OBu)_4_ as catalyst. They obtained a graft copolymer with increased hydrophilicity and controlled degradation rate that may have wide applications in wound dressing and in controlled drug delivery systems. Analogously, Wu *et al*. synthesized a water soluble chitosan derivative grafted with polylactide by ring opening polymerization of d,l-lactide onto chitosan in dimethyl sulfoxide solution, in the presence of triethylamine. The obtained amphiphilic chitosan–polylactide graft copolymer is able to form polymeric micelles. Because of the micelle hydrophobic core they can be used as a promising delivery carrier for the entrapment and controlled release of hydrophobic drugs [167]. 

Graft copolymerization of vinyl monomers onto chitosan using free radical initiation has attracted the interest of many scientists in the last two decades [168]. Grafting with hydroxyethylmethacrylate (MMA) using azobisisobutironitrile (AIBN) [169], methyl methacrylate using Fenton’s reagent as redox initiator [170], dimethylamino ethyl methacrylate and *N,N*-dimethyl-*N*-methacryloxyethyl-*N*-(3-sulfopropyl)ammonium using ceric(IV)salt as redox initiator [171,172], and *N*-isopropylacrylamide by γ−irradiation method [173] have been reported in the literature.

Grafted chitosans have great utility in controlled drug release [174], tissue engineering [175], wound-healing [176] and cardiovascular applications [177,178].

Moreover, there are several reports regarding the use of enzymes in polymer synthesis and modification [179,180]. In fact, enzymes offer the potential advantage of eliminating the hazards associated with reactive reagents and because of their specificity they offer the potential for precisely modifying macromolecular structure to better control polymer function. 

### Chitosan crosslinking

The development of procedures to crosslink cationic polysaccharides has increased their applications. Polymer hydrogels with adequate mechanical properties and high drug loading capability show a big potential as the basis of controlled drug delivery systems [181]. In the case of chitosan the amino groups of the polymer may allow the establishment of different types of interactions with both non-ionic and ionic drugs [182] and also provide pH-sensitive systems, which swell in gastric conditions allowing a site-specific release [183]. Several cross-linking reagents have been used for crosslinking of chitosan such as glutaraldehyde, genipin, ethylene glycol, diglycidyl ether and diisocyanate [184,185,186,187,188]. 

Gupta *et al*. prepared crosslinked chitosan microspheres by dropping a polysaccharide solution into a methanolic solution of NaOH and then adding a solution of glutaraldehyde for crosslinking. They demonstrated that the obtained microspheres were non-toxic and biodegradable, and hence may be considered as suitable candidate for the oral drug delivery [189].

The formation of hydrogels from polymers using non-covalent crosslinking is an useful method to prepare hydrogels for drug delivery. These gels are likely to be biocompatible as gel formation does not require the use of organic solvents or chemical reactions, which may be potentially deleterious to the drug load. Such physically crosslinked chitosan based gels are formed by exploiting either hydrogen bonding or hydrophobic attractions.

The use of tripolyphosphate (TPP) in ionic gelation as a polyanion to cross-link with the cationic chitosan through electrostatic interaction could avoid possible toxicity of reagents used in chemical cross-linking (e.g. glutaraldehyde). TPP cross-linked chitosan beads can be prepared simply by dropping chitosan droplets into TPP solution and this procedure was found to be useful in the pharmaceutical industry [190,191,192,193].

## 7. Composites and hydrogels based on *N*-succinylchitosan/alginate blends

Gomez d’Ayala *et al*. developed a novel calcium sulphate-based system in which calcium sulphate (CHS) can be encapsulated in a polymeric biodegradable and biocompatible matrix, constituted of alginate and *N*-succinylchitosan, in order to retain the structural integrity and decrease the bioresorption rate [194]. 

The hemihydrate form of CHS, better known as Plaster of Paris, has a long history of use as a filling material for bone defects in regenerative techniques [195,196]. Addition of water to CHS powder elicits an exothermic reaction with the end product being the dihydrate form of calcium sulphate (CaSO4·2H_2_O, gypsum). The product as obtained is a paste with good handling properties and mouldability which in several minutes gets a hard cement. In bone regeneration techniques the bone cavity is filled by a mixture of CHS powder and water (or water solution) and the resulting paste is left to harden *in situ* to the dihydrate form [197,198,199]. Despite calcium sulphate efficiency in bone regeneration field, its potential applications are limited by its rapid *in vivo* resorption and brittleness [200].

**Table 3:** Yield strength and Young modulus (± STD) of composites with calcium sulphate and variable amounts of the two polymeric components.

Sample	Yield strength	Young modulus
	(MPa)	(MPa)
CHS/Alg/sCh: 50/10/40	4.58 ± 1.63	427.4 ± 79.8
**CHS/Alg/sCh: 50/20/30**	**13.62** **± 0.41**	**873.9** **± 145.8**
CHS/Alg/sCh: 50/25/25	8.20 ± 0.15	370.8 ± 73.7
CHS/Alg/sCh: 50/30/20	9.02 ± 2.62	437.9 ± 83.5
CHS/Alg/sCh: 50/40/10	3.22 ± 0.59	444.2 ± 74.7
**CHS/Alg/Ch: 50/30/20**	**0.04** **± 0.01**	**9.9** **± 0.6 **

In order to develop a novel calcium sulphate-based composite for bone regeneration applications, *N*-succinylchitosan (sCh), prepared according to an already reported procedure [149], was used in blends with alginate. Finely ground CHS, Alg, and sCh powders were mixed in different compositions and water was added to the mixture. The presence of water promotes the transformation of calcium sulphate from the hemihydrate form to the dihydrate one. Moreover, calcium ions are slowly released from CHS and available for alginate to crosslink. The setting of the materials is due to the transformation of the CHS into the dihydrate form (and partially to the crosslinking of alginate) that leads to the formation of a hard cement. Mechanical analyses in compression mode were performed on composites constituted by a fixed amount of CHS and variable ratios of the other two components and for sake of comparison on a composite in which N-succinylchitosan was substituted by plain chitosan. 

The obtained results, reported in Table 3, demonstrate that the blend of alginate and *N*-succinyl-chitosan causes a substantial reinforcement of composite. It seems that the carboxylic groups grafted onto sCh in addition to those of alginate enhance the chelating power of the polysaccharide mixture because of an additional synergistic effect with respect to the classic ‘‘egg-box’’ effect of alginate. This result is particularly relevant for a given blend composition (namely, 20/30 Alg/sCh).

**Figure 6:**
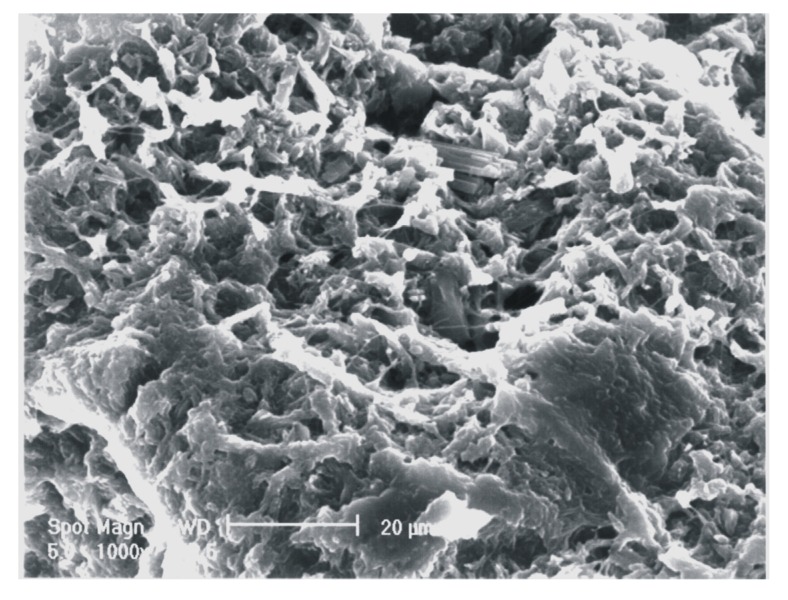
Scanning electron micrographs of CHS/Alg/sCh 50/30/20 (w/w/w) composite*.

Besides, morphological analysis, shown in Figure 6, points out that crystals of CHS are embedded into the polymer matrix with scarce evidence of discontinuity between the polymeric components and the inorganic filler. This could depend on the cooperative effect of chitosan and alginate in crosslinking with calcium ions, that creates a tight polymeric network surrounding gypsum crystals. As matter of fact, in composites in which only one of the two polymers is present, the adhesion between the polymeric and mineral phases notably decreases.

* Reprinted with permission from Gomez d'Ayala *et al*. Development of a new calcium sulphate-based composite using alginate and chemically modified chitosan for bone regeneration. *J. Biomed. Mat. Res. A,* **2007***, 81*, p. 817. Copyright Wiley 2007.

**Figure 7:**
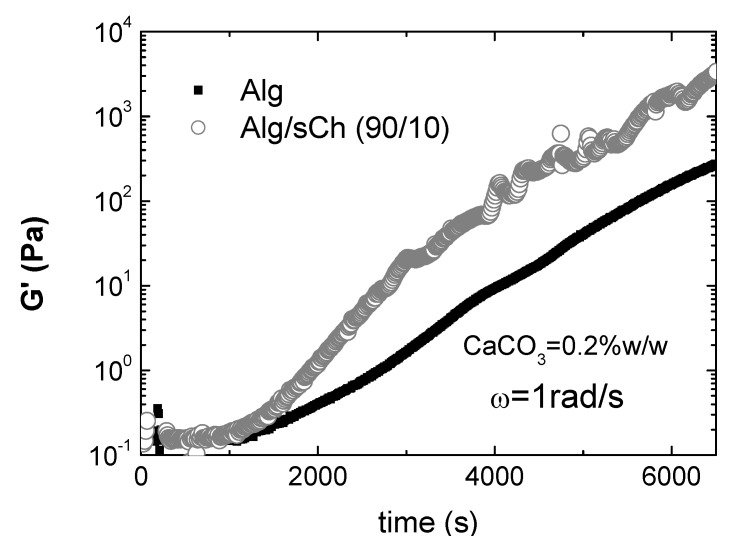
G’ vs. time. Comparison between the plain alginate and the Alg/sCh (90:10) blend (CaCO_3_=0.2% w/w; ω=1rad/s; T=25°C)*.

To investigate in depth such cooperative effects, Nobile *et al*. [201] performed a rheological study on hydrogels of alginate/sCh blends cross-linked via the internal setting method. The calcium carbonate/D-glucono-δ-lactone system was used as calcium ions source to reproduce the condition of slow calcium release, in order to point out the incoming of stronger interactions between the two polymers only for a specific composition. Rheological analyses were carried out by means of oscillatory dynamic measurements. Again, significantly higher viscoelastic parameters were found only for a given composition of the blend (in this case, 90/10 Alg/sCh) This composition may represent a favourable condition at which most of the chitosan molecules are effectively interacting with the alginate network and might co-crosslink with alginate through calcium ions; in turn, this gives rise to a stronger gel, as found. At higher chitosan concentration, the excess of chitosan is bound to the alginate gel network through simple entanglements. Consequently, the final network is partially a ionically cross-linked network and partially a physically entangled network, with a consequent decrease of the viscoelastic parameters, that in some cases are even lower with respect to the plain alginate gel. Besides, it was also demonstrated that a significant acceleration of gelation kinetics occurs for the 90/10 composition, as shown in Figure 7, supporting the hypothesis of a synergistic effect of the N-succinylchitosan in chelating calcium ions during the alginate gelation process. In particular, a value of ~1450s for the induction time and of ~ 2550s for the sol-gel transition have been evaluated for the 90/10 gel, compared to ~ 1800s and ~3470s for the plain alginate gel. This result is quite interesting in view of possible applications as *in situ* gelling systems.

* Reprinted with permission from Nobile *et al*. Development and rheological investigation of novel alginate/N-succinylchitosan hydrogels. *J. Polym. Sci., B: Polym. Phys.* **2008**, *46*, p. 1180. Copyright Wiley 2008.

## 8. Conclusions

It has been shown how the most abundant polymers of marine origin, i.e. alginate, chitin and its derivative chitosan, are employed in many biomedical applications, both alone as well as in composites and in blends, and how their structure can be modified in order to improve deficiencies or to impart them innovative properties. The possibility of producing a variety of chemically modified derivatives makes these polysaccharides versatile biomaterials in almost all fields of biomedical interest. It is possible to forecast that diversified chemical modification approaches will open more and more new perspectives and potential applications in the future. It is also worth mentioning that improving the performance of natural polymers is an opportunity for the medical and pharmaceutical industry, as the time-to-market of the said polymers is reduced, when compared to synthetic biodegradable polymers. The application of the principles of chemical modification to natural polysaccharides will allow for a technological development competitive with that of polymers from petroleum sources.
